# Supplementary figures and images for: Transcriptional Control of Hypoxic Hyphal Growth in the Fungal Pathogen Candida albicans
Source: Front Cell Infect Microbiol. 2022 Jan 19;11:770478. doi: 10.3389/fcimb.2021.770478 (PMC8807691; doi:10.3389/fcimb.2021.770478)

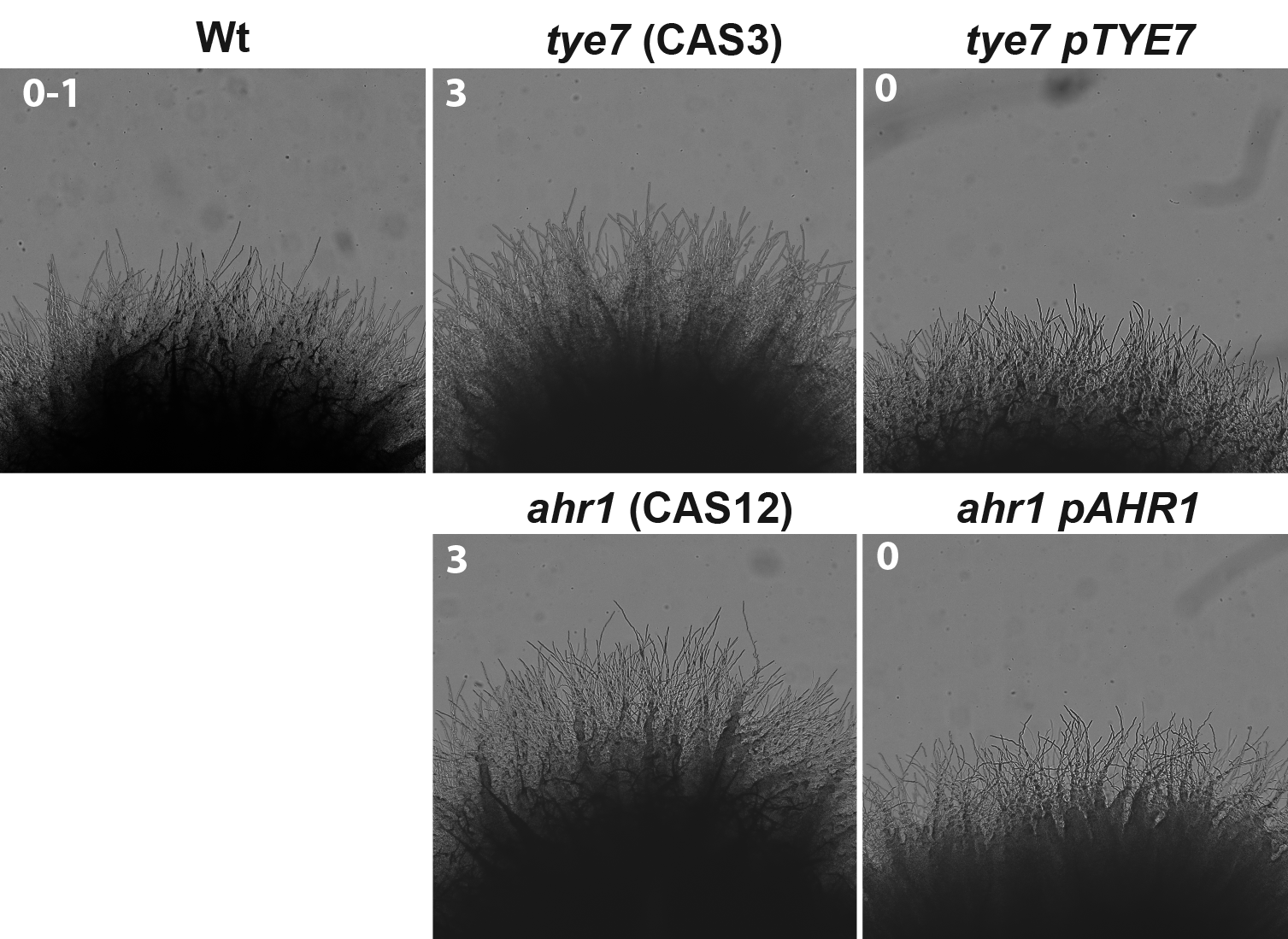

Supplement: Supplementary Figure 1 — Confirmation of ahr1 and tye7 hyperfilamentation under hypoxia in the BWP17 strain background. Complementation of the ahr1 (CAS12) and tye7 (CAS3) mutants with Wt alleles of AHR1 or TYE7 restored the hypoxic filamentation to a level comparable to that of the Wt strain (BWP17). Filamentation scores were indicated for each strain. The C. albicans WT, tye7, ahr1 and the revertant strains were grown on YPS-agar medium and incubated for 6 days at 30°C under hypoxic (1% O2) environments. [file Image_1.tif]

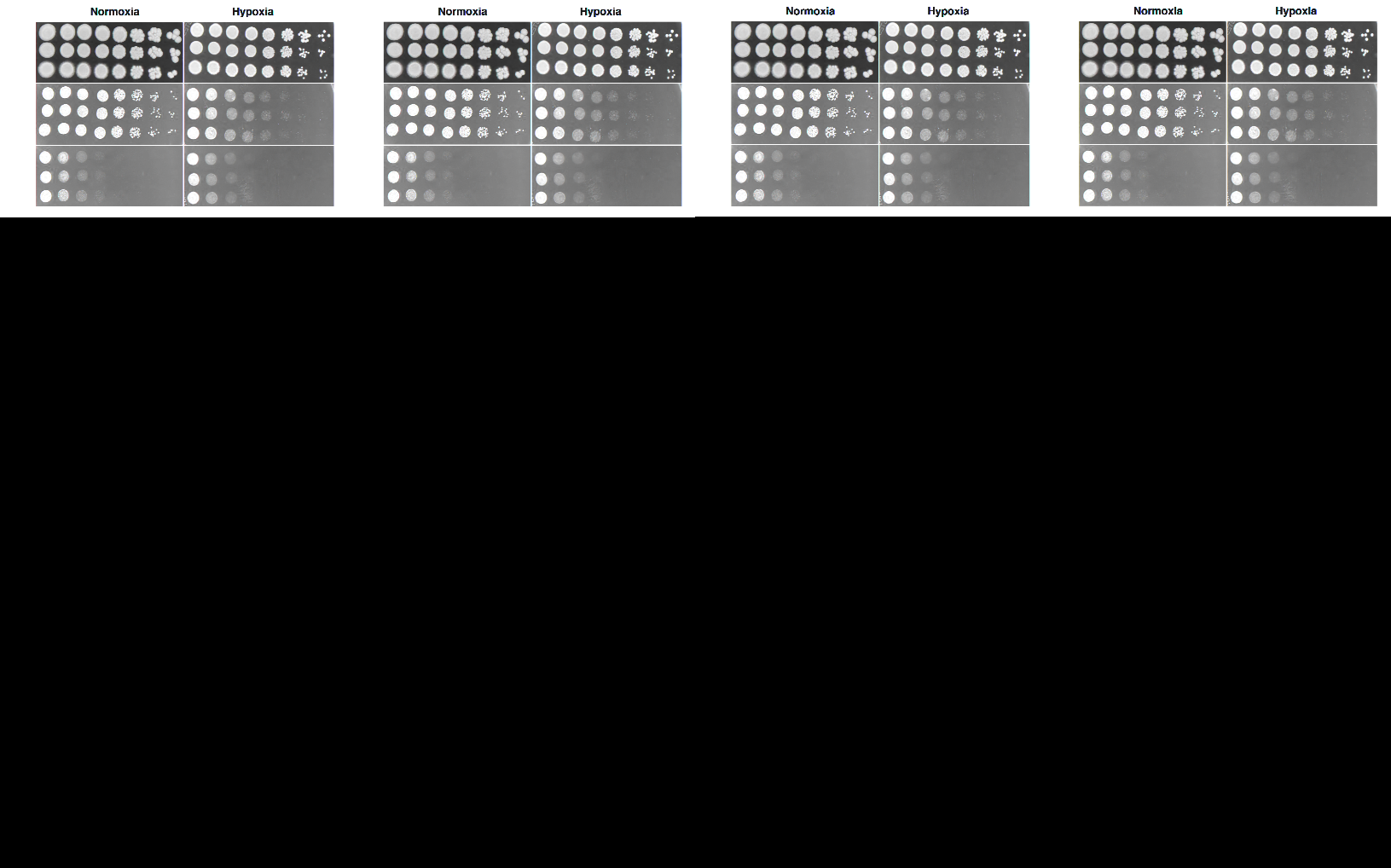

Supplement: Supplementary Figure 2 — The C. albicans Wt, tye7 and ahr1 strains were serially diluted, spotted on YPD-agar medium with different concentrations of BPS and incubated for 2 days at 30°C under normoxic (21% O2) or hypoxic (1% O2) environments. [file Image_2.tif]
